# Differentiating Brain Metastasis and High‐Grade Glioma Using Multi‐*b* Value Diffusion MRI and Tumor Volumetry

**DOI:** 10.1111/jon.70103

**Published:** 2025-11-12

**Authors:** Tereza Kopřivová, Marek Dostál, Tomáš Jůza, Václav Vybíhal, Petra Ovesná, Michal Kozubek, Miloš Keřkovský

**Affiliations:** ^1^ Department of Radiology and Nuclear Medicine, Faculty of Medicine Masaryk University, Brno and University Hospital Brno Brno Czech Republic; ^2^ Department of Biophysics, Faculty of Medicine Masaryk University Brno Czech Republic; ^3^ Department of Neurosurgery, Faculty of Medicine Masaryk University Brno and University Hospital Brno Brno Czech Republic; ^4^ Institute of Biostatistics and Analyses Ltd. Brno Czech Republic; ^5^ Centre for Biomedical Image Analysis, Faculty of Informatics Masaryk University Brno Czech Republic

**Keywords:** classification, diffusion magnetic resonance imaging (MRI), high‐grade gliomas, metastasis

## Abstract

**Background and Purpose:**

To evaluate feasibility of multi‐*b* value diffusion magnetic resonance imaging (MRI) and volumetry in differentiating between brain metastases and high‐grade gliomas (HGGs) while producing a differentiation tool.

**Methods:**

Preoperative brain MRI consisting of both morphological and multi‐*b* value diffusion sequences of patients with HGGs and brain metastases was prospectively performed. Three‐dimensional masks of enhancing and non‐enhancing tumor and surrounding edema were semiautomatically segmented. Multiple diffusion parameters were subsequently derived together with volumes of the particular tissues. Histogram analysis of the diffusion parameters was performed, and the parameters’ diagnostic power to differentiate between the subgroups was evaluated by receiver operating characteristic analysis and least absolute shrinkage and selection operator (LASSO) regression method.

**Results:**

A training dataset included 97 consecutive patients (67 HGGs, 30 metastases), whereas 17 patients (9 HGGs and 8 metastases) comprised a validation group. Overall, 66 histogram diffusion parameters and tissue volumes were found to differ significantly between metastasis and HGG subgroups. LASSO regression identified 17 of these as best predictors. A decision tree using four parameters achieved sensitivity of 90% and 87.5% and specificity of 97% and 77.8% for the training and validation subgroups, respectively.

**Conclusion:**

Multi‐*b* diffusion MRI and tumor volumetry may be valuable diagnostic tools for differentiating HGG from brain metastasis.

## 1 Introduction

The differentiation of high‐grade glioma (HGG) from metastasis using magnetic resonance imaging (MRI) is a challenging yet important task. The etiology of either a primary or metastatic brain tumor influences the planning of further treatment strategies and significantly affects the patient's prognosis [1]. In cases where there is initial suspicion of a brain metastasis, it is advisable to conduct a thorough search for the primary tumor, including advanced methods such as hybrid positron emission tomography and computed tomography [2]. Patients with HGGs more often require neurosurgical resection as an initial approach, whereas cerebral metastases are variably treated with neurosurgical procedures, chemotherapy, immunotherapy, or irradiation, based on the primary tumor and systemic staging [3]. Furthermore, according to studies published in recent years, preoperative stereotactic radiotherapy (SRT) before brain metastasis surgery could achieve higher local control rates with a lower risk of leptomeningeal disease and minimize radiation‐induced tissue damage compared to postoperative SRT [4]. It is also important for the strategy of neurosurgical treatment to know whether the lesion is a brain metastasis or a primary brain tumor. In contrast to HGGs, which are mostly diffusely infiltrating, metastatic lesions are often composed of a dominant tumor mass with generally distinct borders. These lesions tend to displace the surrounding cortex and are surrounded by a gliotic pseudocapsule. This fact is important for surgical planning, especially near eloquent structures. Multiple studies advocate for en bloc resection in the case of brain metastases whenever possible, which is not entirely necessary for malignant gliomas [5].

Distinguishing between brain metastasis and HGG using conventional MRI is often difficult as the lesions have nonspecific patterns and numerous characteristics in common. Because morphological imaging alone has relatively low sensitivity and specificity (e.g., Maurer et al. report accuracy of 68%, sensitivity of 84%, and specificity of 45% [6]), advanced MRI methods are being investigated as a way to improve MRI's diagnostic accuracy.

A number of publications highlight the potential utilization of MR spectroscopy, which has demonstrated significant differences in metabolite concentrations between HGG and brain metastases [7, 8]. Contrast perfusion techniques, such as dynamic susceptibility contrast (DSC), are also promising. These methods have shown increased perfusion values in the perifocal T2 hyperintense zone surrounding HGG compared to edema adjacent to metastases [9], likely serving as a marker of infiltrative spread of non‐necrotic tumor tissue [10]. Additionally, non‐contrast perfusion imaging techniques, such as arterial spin labeling (ASL), have been explored [11]. Among diffusion‐weighted MR imaging techniques, commonly used diffusion‐weighted imaging (DWI) employs a uniexponential model with apparent diffusion coefficient (ADC) calculation, revealing diffusivity changes within the tumor itself or the surrounding T2 hyperintense zone [12]. More advanced diffusion models utilizing measurements with multiple magnetic gradient directions are also applicable. Significant differences in anisotropic diffusion parameters have been demonstrated between HGG and brain metastases using diffusion tensor imaging (DTI) [13]. Recent studies also explore differences in diffusivity between HGG and brain metastases using more advanced “multi‐shell” techniques such as diffusion kurtosis imaging (DKI), neurite orientation dispersion and density imaging (NODDI), or diffusion spectrum imaging (DSI) [14, 15]. These three methods (DKI, NODDI, and DSI) employ higher diffusion weighting values (*b* ≥ 2000 s/mm^2^), which increase the sensitivity of the sequence to water diffusion. However, at such high *b* values, spatial restrictions imposed by biological structures (e.g., cell membranes) become apparent, and diffusion processes begin to exhibit non‐Gaussian behavior. The degree of deviation from Gaussian diffusion can be characterized, for example, by the dimensionless kurtosis coefficient (*K*) [16]. One of the promising diffusion MRI techniques is the intravoxel incoherent motion (IVIM) method. Starting from Einstein's diffusion equation, we can calculate the diffusion coefficient (*D* ≈ 10^−9^ m^2^/s) from the known average velocity of water molecules (*v* ∼ 100 m/s) and their mean displacement (l–10 nm) [16]. Considering blood perfusion (movement of water molecules in blood) as a diffusive process (the so‐called pseudo‐diffusion), using the average blood flow velocity in capillaries (*v* ∼ 1 mm/s) and their mean length (l–100 µm), and applying the same formula, we obtain an approximately tenfold higher pseudo‐diffusion coefficient (D* ≈ 10^−8^ m^2^/s) compared to water diffusion. This difference is sufficiently large to distinguish the two processes but also similar enough to detect both using MRI. Multi‐*b* value DWI enables simultaneous assessment of perfusion‐related pseudo‐diffusion (by analyzing the signal at low *b* values, typically <200 s/mm^2^) and non‐Gaussian diffusion effects reflecting tissue microstructure (by analyzing the signal at high *b* values, typically ≥1500–2000 s/mm^2^) [16]. Given the known differences in perfusion and diffusion between HGG and brain metastasis, we believe that this method holds great potential that has been relatively underreported in the literature [17]. Furthermore, by including images with higher diffusion weighting, it is possible to characterize also the non‐Gaussian behavior of water diffusion through diffusion kurtosis. This approach combines the capability to characterize both diffusion and perfusion within a single acquisition, allowing these two phenomena to be assessed with the same spatial resolution, at the same time, without the need for contrast agent administration, and within a clinically acceptable scan time.

Thus, this study presents a comprehensive diffusion MRI technique that combines IVIM and DKI analysis, which offers several advantages over the aforementioned advanced methods. This approach provides higher spatial resolution compared to ASL and, unlike DSC, does not require the dynamic application of contrast agents. Compared to complex multi‐shell diffusion sequences, it benefits from a shorter acquisition time, whereas the isotropic data obtained allow for the calculation of a range of potentially valuable parameters. The primary objective of this work is to evaluate their effectiveness in distinguishing HGG from brain metastases.

## 2 Methods

### 2.1 Patient Selection

The prospective study was approved by the institutional review board. Within a period of two and a half years (from 2019 to 2023), we enrolled consecutive patients scheduled for surgery for intra‐axial brain lesion with suspicion of HGG or metastases according to the previous imaging performed. Prior to MRI examination, all patients had signed an informed consent form approved by the university hospital ethics committee. We excluded patients whose biopsy result was other than HGG or metastasis, as well as patients with significant motion artifacts on MRI or those whose examinations were incomplete. In the end, we included overall 97 patients with histologically confirmed HGGs or metastases constituting a training subgroup that was consequently analyzed. During the data analysis (first half of 2023), we continued patient recruitment to establish a validation cohort. This cohort ultimately comprised 17 patients with confirmed HGG or brain metastases.

### 2.2 MRI Acquisition

The examinations were performed on a 1.5 Tesla (T) MR device (Philips Ingenia) with 20‐channel head–neck coil. The imaging protocol comprised DWI single‐shot echo planar imaging (SS‐EPI) sequence in axial plane using 10 different *b* values [signal averaging] (0[1], 10[1], 20[1], 30[1], 50[1], 100[2], 200[2], 500[3], 1000[3], 2000[6]), acquisition time 4:50, and sequences for morphological evaluation, including non‐contrast enhanced (ce) two‐dimensional (2D) T1 fast field echo, 2D T2 turbo spin echo (TSE) in axial plane, ce 3D T1 TSE acquisition in axial plane, and three‐dimensional (3D) fluid attenuated inversion recovery (FLAIR) in sagittal plane. The same SS‐EPI DWI sequence with only *b* = 0 and opposite phase‐encoding direction was acquired for susceptibility artifact correction.

### 2.3 Morphological Segmentation

All operations with the images were done by scripts from FSL library [18]. All morphological images (T2, FLAIR, and T1) were registered into T1‐ce space by linear rigid (six degrees of freedom) algorithm (FLIRT, v5.0.10, Oxford, UK, ttps://web.mit.edu/fsl_v5.0.10/fsl/doc/wiki/FLIRT.html) and skull striped by bet (v5.0.10, Oxford, UK, https://fsl.fmrib.ox.ac.uk/fsl/docs/#/structural/bet) [19, 20]. Mean white matter intensities of T1 and T1‐ce images were normalized, and the normalized T1 image was subtracted from the normalized T1‐ce image (resulting in the T1‐sub image). All segmentations were performed by two board‐certified radiologists with more than 6 years’ experience (T.K. and T.J.) using a semiautomatic “classification” algorithm based on decision trees and active contour evolution implemented in ITK‐SNAP software (v3.8.0, Philadelphia, PA, USA, www.itksnap.org) [21]. In the first step, the rater manually delineated the enhancing portion of the tumor (Enh.) and the background on one axial, sagittal, and coronal slice of the T1‐ce and T1‐sub images. Using this initial training segmentation, the ITK‐SNAP algorithm generated a probabilistic map of the enhancing tumor region for the entire lesion. The rater then placed several seed points within the enhancing tumor and initiated active contour evolution. For detailed information on the active contour method, refer to the ITK‐SNAP manual (https://www.itksnap.org/docs/viewtutorial.php?chapter=TutorialSectionRegionSegmentation). In the second step, the tumor core (including both enhancing tissue and non‐enhancing necrotic or cystic part) was segmented on the FLAIR and T1‐ce images using the same approach as described above. The mask of the non‐enhancing tumor portion was obtained by subtracting the enhancing tumor mask from the tumor core mask.

**FIGURE 1:**
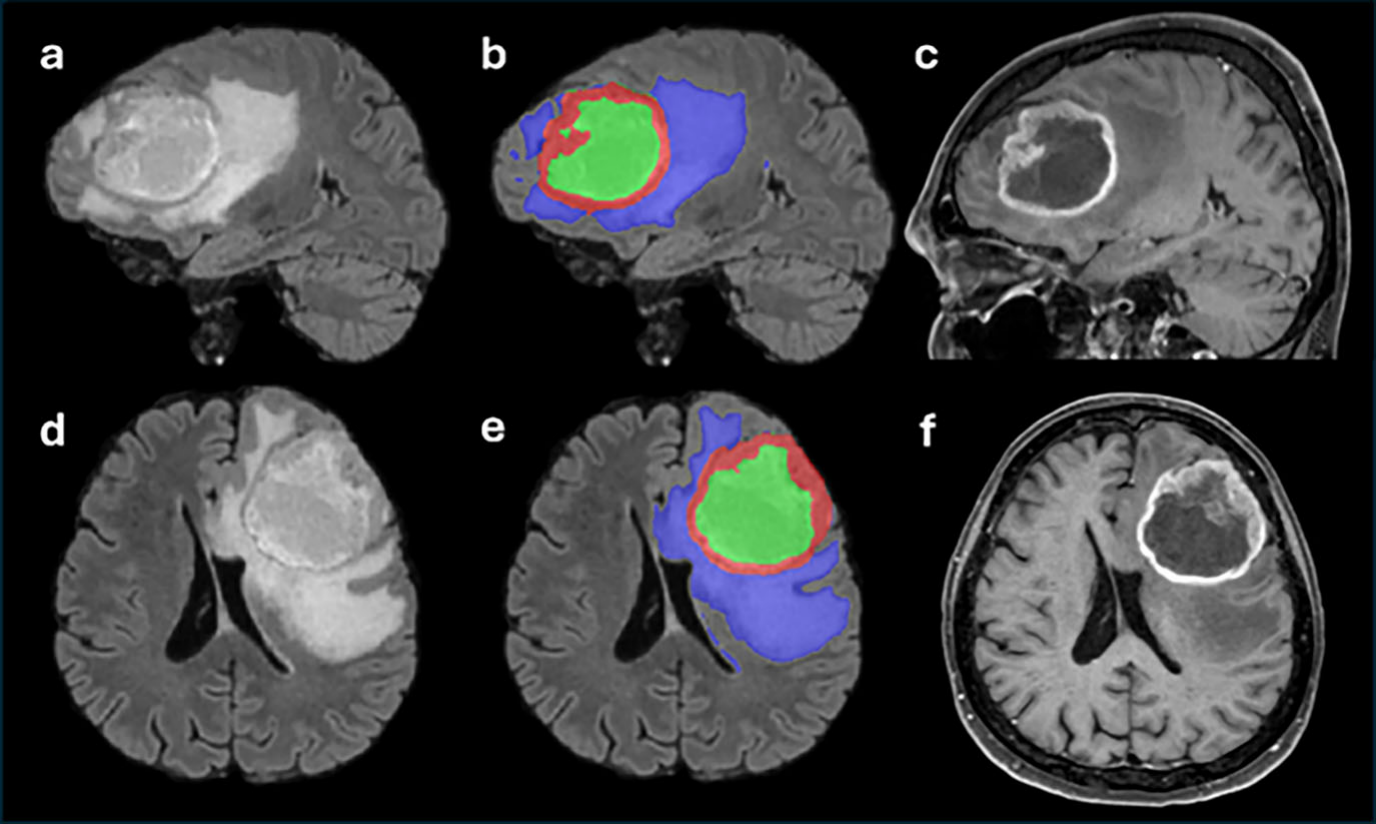
Example of segmentation (b and e) of a high‐grade glioma. The segmentation mask of edema is in blue, enhancing tumor tissue in red, and non‐enhancing (necrotic or cystic) tumor tissue in green. FLAIR images are shown at left anand T1 contrast‐enhanced images at right in sagittal (a and c) and axial (d and f) planes.

In the final step, the entire pathological region was segmented on FLAIR image, including also the surrounding non‐enhancing hyperintense area surrounding the tumor core (hereinafter referred to as edema). The edema mask was calculated by subtracting the tumor core mask from the whole pathology mask. During and after each step, results were visually checked in ITK‐SNAP interface and manually corrected when needed. Finally, all masks were checked for overlapping between tissues, and any overlaps were corrected. Results of this process can be seen in Figure 1. In cases where multiple metastatic lesions were observed in patients, the largest lesion was selected for evaluation, which was subsequently histopathologically confirmed. An inclusion criterion was that this lesion should not be in contact with other mentioned lesions, allowing for clear delineation of all evaluated tissues. Specifically, patients whose multiple enhancing lesions shared an edema zone were excluded from the evaluation.

### 2.4 Multi‐*b* Diffusion Processing

Several preprocessing steps were made using scripts from FSL library (v6.0.5.1, Oxford, UK, https://web.mit.edu/fsl_v5.0.10/fsl/doc/wiki/FLIRT.html). Susceptibility distortion correction was done by the topup algorithm followed by eddy current and movement artifacts correction by the eddy algorithm [22, 23]. Skull striping was done by bet algorithm, and anatomical T2‐w image was registered into *b* = 0 space by linear rigid (six degrees of freedom) registration using the FLIRT algorithm. Three binary masks of segmented tissues were registered into the DWI space by the same registration matrix as for the T2‐w image in the previous step. The first multi‐*b* diffusion analysis was based on the IVIM model which included all *b* values:

(1) $$S\;\left(b\right)=\;S_{0}\left(fe^{-bD*}+\left(1-f\right)e^{-bD}\right)$$

For fitting of IVIM parameters (perfusion fraction [*f*], pseudo‐diffusion coefficient [D*], and diffusion [*D*]), algorithms from the Diffusion in Python (DIPY, v1.4.1, open‐source, https://dipy.org/) [24] library were used together with the optimization process method known as variable projection, which uses a MIX approach [25]. Additional multi‐*b* diffusion analysis was based on a diffusion kurtosis model wherein parameters of diffusion and kurtosis (*K*) were fitted by logarithmic fitting option in Medical Imaging Interaction Toolkit (MITK, v2018.09.99, Heidelberg, Germany, https://github.com/MIC‐DKFZ/MITK‐Diffusion) software with boundaries from 0 to 3, smoothing sigma equal to 5 and using all available *b* values [26, 27]:

(2) $$S\left(b\right)=S_{0}\;e\left(-bD+\frac{b^{2}D^{2}K}{6}\right)$$

**FIGURE 2:**
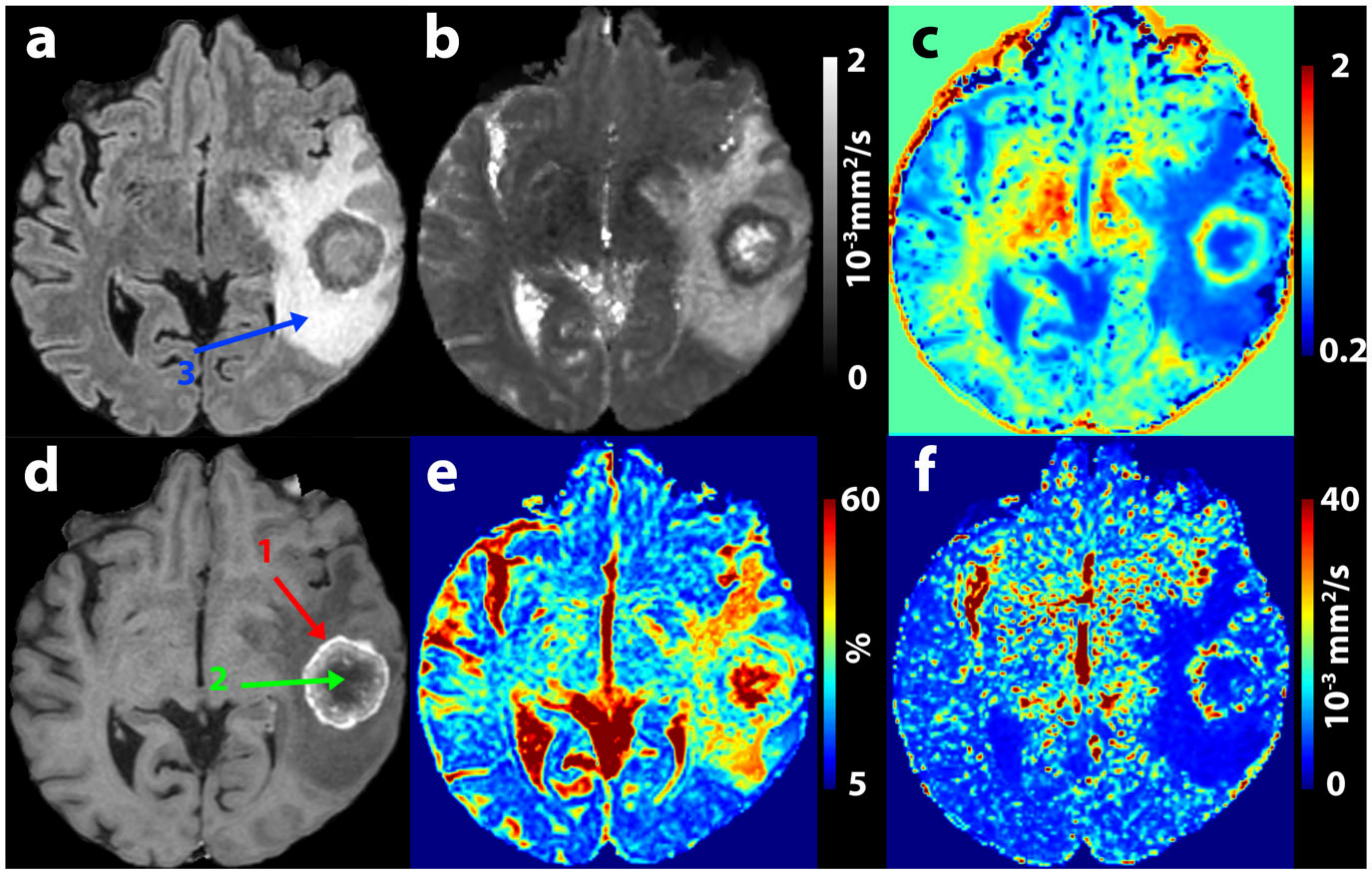
Example of a brain metastasis (female, 44 years old) of colorectal cancer depicted on anatomical sequences in axial plane (a—fluid‐attenuated inversion recovery, d—T1 contrast‐enhanced images) and on calculated multi‐b parametric maps (b—*D*, c—*K*, e—*f*, f—D*). D*—pseudo‐diffusion coefficient, *K*—diffusion kurtosis, 1 (red arrow)—enhancing tumor, 2 (green arrow)—non‐enhancing tumor, 3 (blue arrow)—edema.

For the subsequent statistics, only *D* calculated by IVIM model was used and not the one calculated by the diffusion kurtosis model. An example of multi‐*b* parameter maps is presented in Figure 2.

### 2.5 Statistical Analysis

The two subgroups were compared in terms of age and sex distribution by Student's *t*‐test and chi‐square test, respectively.

We performed histogram analysis to evaluate all voxels belonging to the segmentation masks of particular tissues (enhancing tumor, non‐enhancing tumor, and edema), calculating mean; median; standard deviation; minimum; fifth, 25th, 75th, and 95th percentile values; as well as maximum, skewness, and histogram kurtosis (for kurtosis as a statistical parameter describing the histogram, we will use the term “histogram kurtosis (Kurt.),” whereas for denoting the non‐Gaussian behavior of diffusion, we will use the term simply as “kurtosis (*K*)” or “diffusion kurtosis”) for all parameters (*f*, D*, *D*, and *K*). We also calculated volumes of the segmented tissues. In total, 135 parameters were analyzed.

All parameters were compared between HGG and metastasis subgroups using the Wilcoxon rank sum test with false discovery rate correction for multiple testing. Receiver operating characteristic (ROC) analysis was performed to characterize observed parameters and set optimal thresholds for differentiation between the two subgroups. Odds ratio and 95% confidence interval were calculated for all thresholds by univariate logistic regression models for metastasis prediction. If a specific tissue type was missing from the lesion (edema, enhancing tissue, or non‐enhancing tissue), the value was skipped (32 cases). Furthermore, least absolute shrinkage and selection operator (LASSO) regression method was used to regularize and select variables from the imputed dataset for subsequent multivariable analysis by classification trees. In this case, the missing values were replaced by zero, because LASSO regression method requires numerical input. These analyses were done in R software (https://www.r‐project.org/, Vienna, Austria) using the grpreg v3.4.0 (https://github.com/pbreheny/grpreg), pROC 1.18.5 (https://xrobin.github.io/pROC), and rpart 4.1.21 (https://www.rdocumentation.org/packages/rpart) packages. Significance level was set at *p* < 0.05 for all statistical tests.

Reliability testing of segmentations and the values of histogram parameters were calculated on a subgroup of 14 randomly chosen subjects from the training dataset (7 HGGs and 7 metastases) whose images were segmented independently by both annotators. The agreement of those segmentation results was quantified by intraclass correlation coefficient as calculated by IBM SPSS Statistics 28.0 (v28.0, Armonk, NY, USA, https://www.ibm.com/products/spss‐statistics) for all monitored parameters. Agreement of segmentation masks was evaluated by the Dice coefficient (similarity metric) and averaged Hausdorff distance (distance metric) as calculated by Evaluate Segmentation Tool (v1.0, Vienna, Austria, https://github.com/Visceral‐Project/EvaluateSegmentation) [28].

## 3 Results

The training dataset included 97 patients, 67 with histologically confirmed HGGs and 30 with metastases. Table 1 shows descriptive characteristics of patients included in the training dataset. Metastatic lesions were predominantly solitary (25 cases). The remaining five patients had multiple (n = 2‐7) metastatic lesions; in these cases, only the largest lesion was included in the segmentation mask and used for further evaluation. Frequency of histological grades of HGGs and of primary sites of metastatic lesions are listed in Table 2. The validation dataset included 17 patients (9 HGGs and 8 metastases).

**TABLE 1:** Descriptive characteristics of patients included in the training dataset.

	HGG (*N* = 67)	Meta (*N* = 30)	*p* value
Mean age ± SD	62.36 ± 11.01	60.16 ± 7.97	0.354^a^
Women	29	16	
Men	38	14	0.359^b^

Abbreviations: HGG, high‐grade glioma; Meta, metastasis; N, count of subjects; SD, standard deviation.

^a^ Student's *t*‐test.

^b^ Chi‐square test.

**TABLE 2:** Histological characteristics of lesions included in the training dataset.

Metastases	Lungs	12
	Breast	7
	Malignant melanoma	4
	Kidneys	1
	Ovaries	2
	Tonsils	1
	Thymus	1
	Neuroendocrine tumor	1
	Colon	1
HGGs	WHO gr. III	19
	WHO gr. IV	48

Abbreviations: HGGs, high‐grade gliomas; WHO gr., world health organization grade.

We found 74 histogram parameters overall which differed significantly between HGGs and metastases. Of these, 66 parameters were significant after correction for multiple testing. Table 3 shows median and interquartile range (IQR) values of histogram median values for all multi‐*b* diffusion parameters measured within the particular tissue types together with their volumes.

**TABLE 3:** Basic tissue characterization.

Parameter	Tissue	HGG	Metastases	*q* value
*f*	Enhancing	0.20 (0.18–0.23)	0.23 (0.21–0.25)	0.003*
	Non‐enhancing	0.25 (0.19–0.38)	0.25 (0.20–0.40)	0.704
	Edema	0.20 (0.18–0.23)	0.24 (0.22–0.27)	0.001*
*D* (10^−6^ mm^2^/s)	Enhancing	774.5 (680.75–838)	649 (602.75–700.5)	0.003*
	Non‐enhancing	932 (724–1198)	666 (618–897)	0.023*
	Edema	950 (810–1023)	1012 (905.25–1091)	0.023*
D* (10^−6^ mm^2^/s)	Enhancing	5975.5 (5506–7267.25)	6429 (5576.75–6974)	0.639
	Non‐enhancing	4798 (4436–5128)	5177 (4943–6061)	0.028*
	Edema	5053 (4581–5364)	4611 (4333–4949)	0.016*
*K* ^a^	Enhancing	0.76 (0.68–0.86)	0.91 (0.84–0.99)	0.001*
	Non‐enhancing	0.65 (0.54–0.77)	0.87 (0.70–0.93)	0.02*
	Edema	0.63 (0.59–0.75)	0.62 (0.58–0.70)	0.278
Volume (mL)	Enhancing	11.73 (4.59–23.54)	9.07 (3.94–22.06)	0.796
	Non‐enhancing	8.1 (1.1–25.25)	0.32 (0–5.88)	0.011*
	Edema	39.41 (9.81–67.67)	42.63 (15.7–95.32)	0.572

*Note*: Median values (interquartile range) of all histogram median values for all diffusion parameters measured within particular tissue types separately for high‐grade glioma (HGG) and metastasis subgroups. Significant results of Wilcoxon rank sum test with false discovery rate correction for multiple testing (*q* values) are bold. *f*—perfusion fraction, *D*—diffusion coefficient, D*—pseudo‐diffusion coefficient, *K*—kurtosis, *—statistically significant values.

^a^ Dimensionless.

Table 4 shows results of ROC analyses and linear regression demonstrating the power of the 10 most significant histogram parameters (maximum sum of sensitivity and specificity) measured within each tissue to differentiate between the subgroups.

**TABLE 4:** Ten most significant parameters for each tissue based on receiver operating characteristic results and regression analyses.

	*D*. par.	Histogram par.	Threshold	AUC (95% CI)	Sensitivity	Specificity	OR (95% CI)	*p* value
Enhancing	*K*	95%	1.243	0.822 (0.726–0.918)	0.667	0.877	14.25 (5.15–43.59)	<0.001
	*K*	75%	0.975	0.817 (0.726–0.909)	0.8	0.738	11.29 (4.16–34.97)	<0.001
	*D*	25%	588	0.795 (0.69–0.899)	0.767	0.754	0.099 (0.034–0.263)	<0.001
	*D*	5%	470	0.806 (0.704–0.909)	0.734	0.785	0.099 (0.035–0.262)	<0.001
	*K*	Mean	0.81	0.811 (0.721–0.9)	0.867	0.646	11.87 (4.038–44.03)	<0.001
	*D*	Median	718	0.747 (0.639–0.855)	0.834	0.662	0.10 (0.031–0.284)	<0.001
	*f*	25%	0.162	0.739 (0.627–0.851)	0.667	0.815	8.83 (3.402–24.65)	<0.001
	*f*	Median	0.209	0.737 (0.632–0.843)	0.8	0.646	7.30 (2.75–22.11)	<0.001
	*f*	Skewness	1.047	0.737 (0.635–0.84)	0.834	0.585	0.14 (0.044–0.391)	<0.001
	*f*	Kurtosis	0.839	0.706 (0.6–0.811)	0.834	0.569	0.15 (0.046–0.416)	<0.001
Non‐enhancing	*D*	Mean	770	0.699 (0.543–0.856)	0.7	0.774	0.13 (0.037–0.38)	<0.001
	*K*	Median	0.797	0.715 (0.569–0.861)	0.7	0.774	7.97 (2.626–26.99)	<0.001
	*K*	75%	0.967	0.731 (0.584–0.878)	0.65	0.811	7.99 (2.623–26.601)	<0.001
	*D*	75%	887	0.681 (0.522–0.84)	0.7	0.755	0.14 (0.042–0.419)	<0.001
	D*	25%	4564	0.711 (0.559–0.863)	0.6	0.849	8.44 (2.71–28.63)	<0.001
	D*	Median	5105	0.698 (0.549–0.847)	0.7	0.736	6.5 (2.18–21.594)	0.001
	*D*	25%	583	0.71 (0.56–0.86)	0.6	0.83	0.14 (0.041–0.417)	<0.001
	D*	Skewness	3.02	0.714 (0.568–0.86)	0.65	0.774	0.16 (0.049–0.469)	0.001
	*K*	95%	1.176	0.707 (0.561–0.852)	0.65	0.774	6.35 (2.13–20.513)	0.001
	*D*	95%	1073	0.689 (0.529–0.85)	0.6	0.811	0.16 (0.048–0.467)	0.001
Edema	*f*	25%	0.147	0.808 (0.72–0.896)	0.893	0.651	15.53 (4.784–70.52)	<0.001
	*f*	Median	0.197	0.786 (0.693–0.88)	0.964	0.540	31.66 (6.14–581)	<0.001
	D*	Mean	6941	0.728 (0.61–0.847)	0.857	0.587	0.12 (0.032–0.346)	<0.001
	D*	SD	4822	0.729 (0.605–0.853)	0.679	0.762	0.15 (0.053–0.384)	0.001
	*f*	75%	0.255	0.746 (0.642–0.85)	0.929	0.508	13.42 (3.59–87.77)	<0.001
	*f*	Skewness	1.056	0.738 (0.635–0.841)	0.929	0.444	0.09 (0.015–0.36)	0.003
	D*	95%	17,454	0.736 (0.614–0.858)	0.75	0.651	0.18 (0.062–0.468)	<0.001
	D*	25%	4477	0.675 (0.561–0.79)	0.929	0.444	0.09 (0.015–0.36)	0.003
	*K*	Kurtosis	3141	0.656 (0.536–0.777)	0.643	0.73	4.87 (1.919–13.05)	0.001
	D*	Skewness	4.459	0.71 (0.588–0.832)	0.643	0.714	4.5 (1.78–11.98)	0.002

Abbreviations: AUC, area under the curve; CI, confidence interval; D*, pseudo‐diffusion coefficient in (10^−6^ mm^2^/s); *D*, diffusion coefficient in (10^−6^ mm^2^/s); *D*. par., multi‐*b* DWI parameter; *f*, perfusion fraction; *K*, diffusion kurtosis (non‐Gaussian behavior); OR, odds ratio; SD, standard deviation.

**FIGURE 3:**
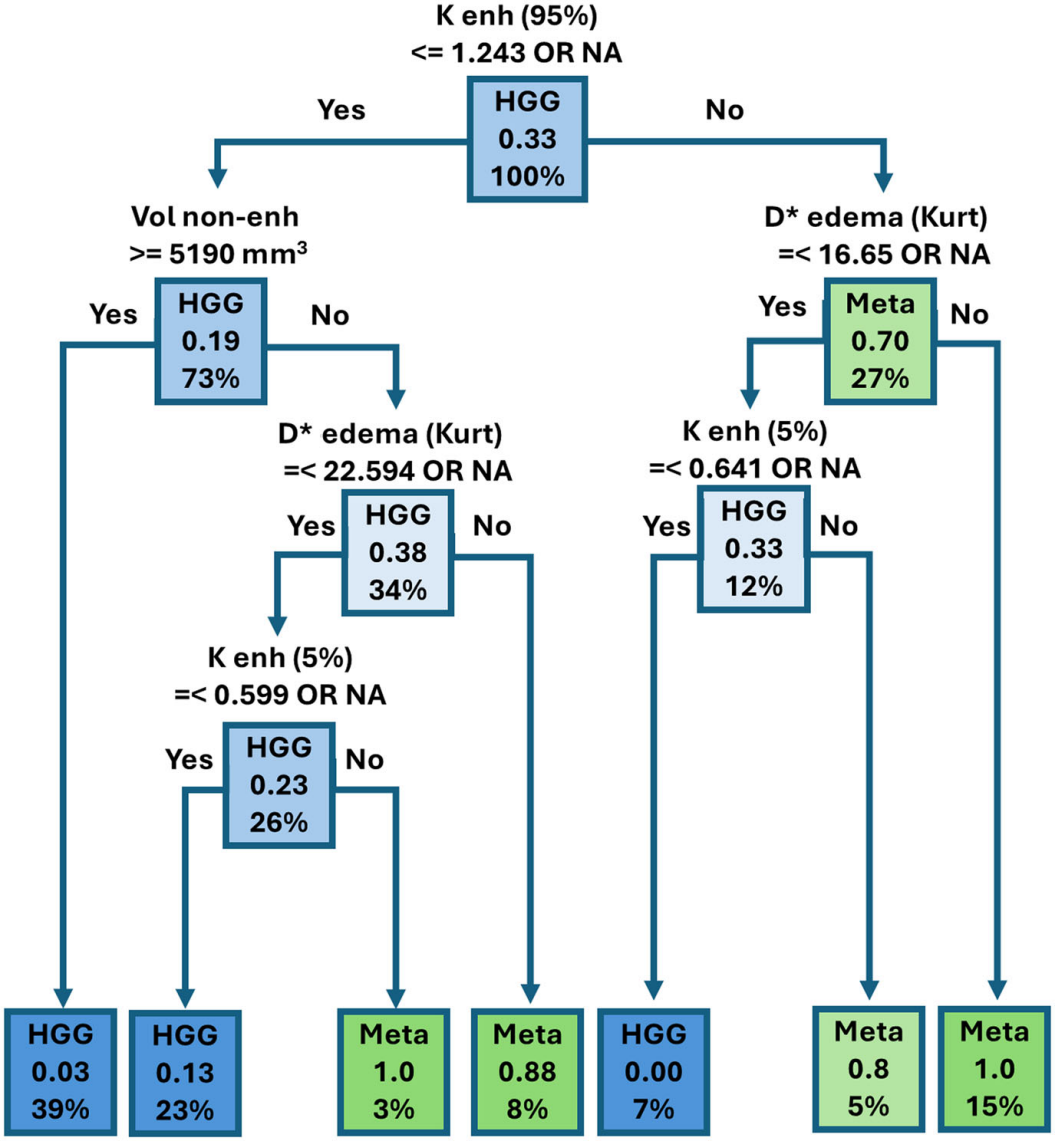
Result of meta/high‐grade glioma (HGG) decision tree analysis. Predicted class (metastasis or HGG) for a particular combination of factors. *x*.*xx*—actual proportion of patients with metastasis in each subgroup according to the combination of selected factors, *x*%—percentage of patients in each subgroup by combination of factors (sum of percentages in each layer gives 100%). *K* enh 95%–95th percentile of diffusion kurtosis in enhancing tissue, Vol non‐enh—volume of non‐enhancing tissue, D* edema Kurt—histogram kurtosis of pseudo‐diffusion in edema, *K* enh 5%—fifth percentile of diffusion kurtosis in enhancing tissue.

LASSO regression identified 17 parameters as the best predictors. On the basis of these parameters, decision tree analysis was performed. The final decision tree included four diffusion histogram parameters and volume of non‐enhancing tissue with specific thresholds, providing 90% sensitivity and 97% specificity with area under curve (AUC) 0.977 for differentiating HGG from metastasis (Figure 3).

**FIGURE 4:**
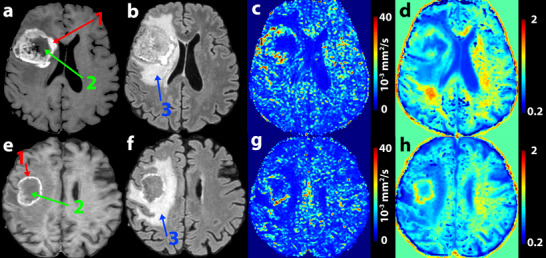
Example of two patients from the decision tree test cohort. The top row shows a patient (male, 67 years old) with a high‐grade glioma grade IV (HGG), and the bottom row shows a patient (male, 73 years old) with a colorectal carcinoma metastasis. On the post‐contrast T1‐weighted images (a and e), the enhancing (red arrow, Label 1) and non‐enhancing (green arrow, Label 2) tumor portions are visible. On the fluid‐attenuated inversion recovery (FLAIR) images (b and f), the edematous region (blue arrow, Label 3) can be seen. Only the multi‐*b* value maps that are used in the decision tree are displayed: pseudo‐diffusion (D*) (c and g) and diffusion kurtosis (*K*) (d and h). The median values are as follows: median D* in edema (10^−6^ mm^2^/s)—HGG = 4938, metastasis = 4544; median *K* in enhancing part—HGG = 0.822, metastasis = 0.927. Values used for decision tree evaluation include as follows: 95th percentile of *K* in enhancing region—HGG = 1.1103, metastasis = 1.3432; histogram kurtosis of D* in edema—HGG = 15.1931, metastasis = 34.4859; volume (cm^3^) of non‐enhancing tumor part—HGG = 13.246, metastasis = 12.221; fifth percentile of *K* in enhancing region—HGG = 0.5903, metastasis = 0.6538.

The diagnostic power of the decision tree was verified on the testing group of 17 patients, achieving sensitivity of 87.5% (7 out of 8 metastatic patients were classified correctly) and specificity of 77.8% (7 out of 9 HGG patients were classified correctly) with AUC 0.778. The testing results are shown in Table 5 and Figure 4.

**TABLE 5:** Results of decision tree validation on training dataset.

		Histopathology	
		HGG	Metastasis
Prediction	HGG	7	1
	Metastasis	2	7

*Note*: Number of patients = 17.

Abbreviation: HGG, high‐grade glioma.

### 3.1 Reliability

Masks of all 14 evaluated subjects included edema, 13 included enhancing tumor, and 12 included non‐enhancing tissue. Similarity and distance metrics describing the agreement of two different readers are shown in Table 6.

**TABLE 6:** Mean values of dice and average Hausdorff distance of segmentation masks done by 2 raters on 14 patients.

Tissue	Dice	Average Hausdorff distance (mm)
Enhancing	0.912 ± 0.065	0.081 ± 0.109
Edema	0.849 ± 0.098	0.428 ± 0.579
Non‐enhancing	0.701 ± 0.245	0.719 ± 0.818
Whole pathology	0.926 ± 0.028	0.073 ± 0.039

*Note*: All the data represent mean ± standard deviation.

Reliability of selected diffusion‐related parameters included in the decision tree is shown in Table 7.

**TABLE 7:** Results of reliability by intraclass correlation coefficient of parameters used in decision tree.

Parameter	Tissue	Histogram	ICC (LB–UB)	*p* value
*K*	Enhancing	95%	0.999 (0.995–1.000)	<0.001
Volume	Non‐enhancing		0.990 (0.965–0.997)	<0.001
D*	Edema	Kurtosis	0.828 (0.487–0.944)	0.001
*K*	Enhancing	5%	0.992 (0.974–0.997)	<0.001

Abbreviations: D*, pseudo‐diffusion coefficient; ICC, intraclass correlation coefficient; *K*, diffusion kurtosis (non‐Gaussian behavior); LB, lower bound; UB, upper bound.

## 4 Discussion

We found several histogram diffusion parameters that differed significantly between those patients with HGG and with brain metastasis. Significantly different, too, was the volume of the non‐enhancing tumor tissue. Among 66 parameters that differed significantly between the subgroups, we observe several median values to be most illustrative for interpretation and comparison to the existing literature data. Perfusion fraction *f*_med_ was significantly higher in enhancing tissue of metastases, which accords with the rather sparse literature to date concerning multi‐*b* diffusion imaging in this application [29]. This finding is mostly inconsistent, however, with those from studies investigating conventional perfusion imaging, as these have found the vascularity of enhancing tumor to be significantly greater in HGGs than in metastases [9]. This result may indicate that perfusion parameters obtained by multi‐*b* imaging do not fully correlate with those obtained by conventional perfusion imaging methods, although general correlation of IVIM parameters with MRI perfusion has been previously reported [16]. Furthermore, the results might be influenced by the heterogenous vascularity of metastatic lesions, depending on their origin, as it has been shown by a study using DSC that metastases of malignant melanoma, for example, have similarly high perfusion as do HGGs [30]. Similarly, diffusivity parameters may differ between different types of brain metastases; Meyer et al. demonstrated, in their study evaluating diffusion‐weighted MRI images, significant differences in ADC values between brain metastases originating from small‐cell and non‐small‐cell lung carcinomas [31]. In another study, significant differences in ADC values were reported between well‐differentiated and poorly differentiated adenocarcinomas [32].

Greater structural complexity of metastatic tissues may present a histopathological background for significantly higher median kurtosis (*K*_med_) and lower median diffusion (*D*_med_) values in enhancing tumor within the metastasis group compared to the HGG group.

Within the edema, significantly higher values of D*_med_ in HGGs may reflect greater perfusion related to the content of tumor infiltration within the T2 hyperintense area surrounding the enhancing tumor [33]. However, the other perfusion‐related parameter (median *f* [*f*_med_]) was higher in edema within the metastases group, which, although not correlated with lower D*_med_ values, is in line with findings of a previously published study employing multi‐*b* diffusion [34]. A possible explanation can lie in the fact that *f* is inherently affected by the overall diffusivity within edema, as reflected by higher *D*_med_ in edema within the metastasis group. Lower *D*_med_ values measured in edema within the HGG subgroup may also indicate a greater cellular density due to the tumor infiltration spreading beyond the boundary of the enhancing lesion, as has been suggested by numerous studies investigating conventional DWI and DTI imaging [35].

*D*_med_ values in non‐enhancing tumor were higher, and D*_med_ and *K*_med_ were lower in the HGG compared to the metastases group. This might be explained by a higher degree of structural disintegration within a necrotic tissue in HGGs, which leads to lower tissue complexity (lower *K*_med_ and higher *D*_med_) and less perfusion (lower D*_med_). Additionally, the volume of non‐enhancing tumor tissue was significantly greater in HGGs compared to metastases. All these findings generally demonstrate a higher degree of necrosis in HGGs, which accords with studies evaluating morphological MR images [36] and DTI [37].

Figure 2 demonstrates a typical multi‐*b* value DWI finding in a patient with metastasis. The contrast‐enhancing tumor tissue shows strikingly low *D* values, as well as comparatively high *f* and *K* values. Within the edema surrounding the lesion, relatively high values of *f* and markedly low values of D* are evident. Comparison of an HGG and a metastatic patient from the test cohort, both correctly classified by the decision tree, is shown in Figure 4. In the edema region, differences in the homogeneity of D* are evident, with areas of higher values observed in the HGG, whereas the edema surrounding the metastasis appears more homogeneous. This is also reflected in the shape of the histogram and the differing values of histogram kurtosis.

For differentiation between the two subgroups (to distinguish metastasis from HGG), we can, based on ROC results, roughly characterize the individual tissues as follows: Analysis of multi‐*b* parameters in edema provides comparatively high sensitivity, non‐enhancing tissue is more likely to contribute with high specificity, and enhancing tissue is balanced between sensitivity and specificity. Appropriate combination of parameters calculated by LASSO analysis and used in the final decision tree achieved high sensitivity and specificity on the training group (90% and 97%, respectively) with only slightly poorer results achieved in the validation group.

A number of studies utilizing various advanced MRI techniques have been published on the topic of HGG classification and brain metastases. According to the meta‐analysis by Suh et al., the combination of DWI and DTI brings pooled sensitivity of both DWI and DTI to 79.8% and the pooled specificity to 80.9% which is comparable to our results [38].

Study by Tan et al. [39] showed that DKI together with directional analysis could lead to improved differentiation with better sensitivity and directional specificity than DTI. The AUC values of corrected axial *K*, mean kurtosis (MK), and radial *K* were 1.000, 0.889, and 0.880, respectively. A recent study by Bai et al. [40] examining the NODDI radiomics model demonstrated that the model was better than the DWI and DTI models, mainly within the solid tumor regions, where it demonstrated superior performance (AUC = 0.904). The multiparametric classification tree we designed demonstrated a high AUC value for the training cohort (0.977) and a relatively lower value for the testing cohort (0.778). However, it is important to note that the assessment of diagnostic utility on the testing dataset may be influenced by a comparatively smaller number of subjects included in testing group. It should also be noted that the mentioned study employed more complex methodological approaches, including radiomics and time‐consuming diffusion data acquisition for NODDI analyses. Meta‐analysis by Usinskiene et al. [41] showed that perfusion and spectroscopy parameters of MR spectroscopy cannot reliably distinguish between HGG and brain metastases.

Another meta‐analysis by Mohammadi et al. [42], which included studies using dynamic contrast‐enhanced (DCE), DSC, and ASL, also showed that perfusion‐derived parameters cannot reliably distinguish between brain metastases and HGG; however, the ASL method yielded slightly more promising results. On the other hand, a different meta‐analysis highlights the significance of the relative cerebral blood volume parameter measured within the peritumoral region, especially when combined with the FA parameter derived from DTI data [9]. Thus, the conclusions of studies using the DSC technique for this classification are somewhat inconsistent.

Although contrast agent administration in our study is used for visualization of the tumor's non‐necrotic tissue and subsequent segmentation, the application of contrast agents via injector in two doses for extracellular space presaturation, as required by DSC [43], is not necessary. The described IVIM technique is therefore advantageous in terms of simpler technical implementation, reduced costs, and decreased risk of potential complications, such as paravenous contrast administration during high‐rate injections. Moreover, DCE or DSC imaging requires sufficient temporal resolution, which often comes at the expense of spatial resolution, signal‐to‐noise ratio, or anatomical coverage, potentially leading to incomplete assessment of the entire pathology. Another advantage of multi‐*b* technique is the ability to quantify additional diffusion parameters, including commonly used ADC values, which would require an additional separate acquisition of diffusion‐weighted sequence if DSCs were used.

We recognize several limitations to the presented study:

We used a 1.5T MR system, which offers lower signal‐to‐noise ratio in comparison with 3T systems. On the other hand, 1.5T systems are more widely used in clinical practice and are less prone to susceptibility artifacts, which can be distinct especially close to bone, air, or hemorrhage.

To identify the area from which to analyze the multi‐*b* diffusion data, we used semiautomatic segmentation of 3D masks of enhancing tumor, non‐enhancing tumor, and peritumoral edema. This approach was based on that of the federated tumor segmentation tool [44]. This method has been reported sporadically in the literature published to date, as most of the previous studies used 2D regions of interest (ROIs) situated in different areas and tumor tissues. In our study, we instead used 3D segmentation together with analysis of histograms of the diffusion parameters, as this approach could provide more comprehensive results than would analysis of 2D ROIs [45]. On the other hand, the methodological difficulty, time demands, and to some extent reliability of segmentation process can be viewed as weak points of this approach. Although our raters achieved high consistency in terms of agreement on the segmentation masks and values of the diffusion parameters themselves, they were trained in how to use ITK‐SNAP software simultaneously. Therefore, the new rater with less intensive training or less experience with MRI image segmentation might achieve lower accuracy. In this context, the employment of fully automatic segmentation using machine learning [46] might bring even higher reproducibility and could also accelerate the whole workflow. In any case, our work achieved similar results despite its methodological approach being different from that of a previous study using manual placement of 2D ROIs [34]. This demonstrates reproducibility of the multi‐*b* diffusion imaging itself, despite the differences in methodology.

Another limitation is generally inherent to the methodology of multi‐*b* data acquisition and analysis. The calculated parameters may not be entirely reproducible and comparable to those from other studies due to the impact of differences in the setting of acquisition parameters, especially the number and distribution of *b* values [46]. Furthermore, differences in data postprocessing, namely, application of different curve‐fitting models for diffusion data, generally have significant impacts on a method's capability to differentiate between different tissues and lesions [47, 48]. Regarding the postprocessing of multi‐*b* diffusion data, we decided to split them into two parts (IVIM model [Equation 1] and diffusion kurtosis model [Equation 2]) and used generally accepted algorithms implemented in DIPY and MITK. The DIPY library offers two different approaches for fitting IVIM parameters, one of which is a “two‐step” approach, which requires that the operator set a specific *b* values threshold for the calculation of *D*, *f*, and D* parameters. In as much as we wanted to minimize human input, we choose the “one‐step” approach instead, and this does not require a threshold setting (“variable projection” method). This method provides more stable results but requires more computing time [25]. Although it is rather difficult to find a study in the literature addressing calculations of non‐tensorial kurtosis data, MITK is one of a few freeware software applications that is generally accepted for DWI analyses and provides reliable output, as demonstrated in previous work [49]. In this study, we chose to separate the IVIM model and the diffusion kurtosis model. However, some publications utilize a combined equation for the IVIM‐DKI model [49, 50, 51]. As suggested by other works [48, 52, 53, 54], the use of this composite model requires a larger number of *b* values to ensure reproducible results. To date, findings in this area remain inconclusive.

Loução et al. [50] also directly compared *K* and MK parameters and *D* and mean diffusivity (MD) parameters, respectively, and these were highly correlated mainly in the edema and tumor regions (Spearman's *ρ* ≥ 0.68). Thus, it seems that non‐tensorial parameters (*D* and *K*) calculated from multi‐*b* diffusion data might be roughly comparable to MD and MK derived from multidirectional diffusion measurements. Because the aforementioned study dealt specifically with HGG and LGG patients, these assumptions cannot be generalized as well as are not directly comparable to those of our study.

A certain limitation of the analysis method is the insertion of zero values when a particular tissue was absent. This step is necessary for the employed LASSO analysis, which requires numerical input. The motivation for this approach was to incorporate information about missing tissue, which can itself serve as a valuable hint toward accurate diagnosis. On the other hand, it should be noted that this solution introduces some bias into the overall structure of the classification tree with regard to the selection of appropriate parameters. The threshold values within the presented decision tree, however, are not affected by this issue in the same way as other analyses, where missing values were simply omitted. Consequently, the reported descriptive statistics and ROC analyses reflect the diffusivity characteristics solely of tissues that are actually present.

Another drawback of this study might be seen in the disbalance between numbers of subjects in the HGG and metastasis groups. Although this may impact upon the statistical power of the analyses, this situation could not really be eliminated in as much as we insisted on histopathological confirmation. Although surgery is the dominant treatment option in patients with HGG, patients with brain metastasis, and especially those with multiple lesions, are treated nonsurgically, so the number of patients with histologically confirmed brain metastasis is naturally limited.

It is also important to mention the heterogeneity of the patient cohort with brain metastases, predominantly originating from primary lung and breast tumors. Given the known differences in diffusivity and perfusion among various histopathological types of brain metastases, one can expect less robust classification accuracy for metastases from less common tumor types or those not represented in our dataset. An additional interesting avenue for future research would be a comparison of multi‐*b* value imaging data across individual metastatic lesions in the brain, which would, however, require a larger patient cohort.

Finally, our study protocol did not encompass conventional DSC perfusion MRI. The direct correlation of this modality with IVIM parameters might further elucidate and support the findings and may be a subject of further studies.

## 5 Conclusion

Analysis of multi‐*b* diffusion data combined with tumor volumetry has potential to improve diagnostic accuracy in differentiating between metastases and HGGs. The presented differentiation tool achieved sensitivity of 90% and 87.5% and specificity of 97% and 77.8% for differentiating metastasis from HGG in training and validation subgroups, respectively. Validation on larger datasets as well as investigation as to impacts of various methodological approaches are needed. Although the presented methodology is currently somewhat challenging and time‐demanding, it can be expected that the introduction to this field of new methods using artificial intelligence may significantly facilitate the use of such complex analysis of the diffusion data.

## Funding

This study was supported by Ministry of Health, Czech Republic— grant number NU21‐08‐00359 and conceptual development of research organization (FNBr, 65269705).

## Conflicts of Interest

The authors declare no conflicts of interest.
